# Effects of Non-Surgical Periodontal Treatment on Reactive Oxygen Metabolites and Glycemic Control in Diabetic Patients with Chronic Periodontitis

**DOI:** 10.3390/antiox10071056

**Published:** 2021-06-30

**Authors:** Simone Marconcini, Enrica Giammarinaro, Saverio Cosola, Giacomo Oldoini, Annamaria Genovesi, Ugo Covani

**Affiliations:** 1 Department of Stomatology, Tuscan Stomatologic Institute, Foundation for Dental Clinic, Research and Continuing Education, 55041 Camaiore, Italy; simosurg@gmail.com (S.M.); s.cosola@hotmail.it (S.C.); giacomo.oldoini88@gmail.com (G.O.); anm.genovesi@gmail.com (A.G.); covani@covani.it (U.C.); 2 Department of Dentistry, Unicamillus International Medical University, 00100 Rome, Italy

**Keywords:** oxidative stress, diabetes, lactoferrin, ozone, periodontal health, reactive oxygen metabolites

## Abstract

Periodontal infection may contribute to poor glycemic control and systemic inflammation in diabetic patients. The aim of the present study is to evaluate the efficacy of non-surgical periodontal treatment in diabetic patients by measuring oxidative stress outcomes. Sixty diabetic patients with periodontitis were enrolled, treated with scaling and full-mouth disinfection, and randomly prescribed chlorhexidine mouthwash, antioxidant mouthwash, or ozone therapy. Reactive oxygen metabolites (ROMs), periodontal parameters, and glycated hemoglobin were measured at baseline and then at 1, 3, and 6 months after. At baseline, all patients presented with pathologic levels of plasmatic ROM (388 ± 21.36 U CARR), higher than the normal population. Probing depth, plaque index, and bleeding on probing values showed significant clinical improvements after treatment, accompanied by significant reductions of plasma ROM levels (*p* < 0.05). At the 6-month evaluation, the mean ROM relapsed to 332 ± 31.76 U CARR. Glycated hemoglobin decreased significantly (∆ = −0.52 units) after treatment. Both the test groups showed longer-lasting improvements of periodontal parameters. In diabetic patients, periodontal treatment was effective at reducing plasma ROM, which is an indicator of systemic oxidative stress and inflammation. The treatment of periodontal infection might facilitate glycemic control and decrease systemic inflammation.

## 1. Introduction

Diabetes mellitus is a major health issue throughout the world. The human and economic burden of diabetes is due to its increasing incidence (439 million people will be affected by 2030) and its numerous complications [1]. Periodontal disease is one of the six major complications of diabetes mellitus [2]. Both diabetes and periodontitis have an inflammatory basis and are linked by different biochemical and metabolic interactions [3]. There is increasing evidence that systemic inflammation resulting from the entry of oral microbiota and their virulence factors into the circulation could impair the course of other co-morbidities, such as diabetes and cardiovascular disease [4,5,6]. It has been suggested that periodontal therapy may improve insulin sensitivity by reducing peripheral inflammatory cytokine levels [7,8]. A significant improvement of glycemic status, defined as a reduction of glycated hemoglobin (HbA1c) of 0.4 units, was demonstrated in diabetic patients suffering from periodontitis after non-surgical periodontal treatment (2013 Joint EFP/AAP Workshop on Periodontitis and Systemic Diseases) [9].

However, there is still a lack of clarity regarding the consequences of untreated periodontal disease in diabetic patients. Furthermore, there are some diabetic patients that do not develop periodontal disease; therefore, it could be of great clinical interest to gain deeper knowledge of the individual protective factors in order to design more accurate diagnostic and therapeutic protocols [10,11].

Several epidemiological studies have reported higher levels of reactive oxygen species (ROS) in the blood of diabetic patients, and the chronic release of ROS is a distinctive trait in the two-way relationship between diabetes and periodontal disease [12,13,14]. There is evidence that oxidative stress is a major cause of diabetic complications [15]. In the endothelial cells, the excessive load of glucose triggers the formation of ROS, which, in turn, impairs mitochondrial function [16]. Due to high reactivity, the ROS reacts with different cellular components, such as DNA, lipids, and proteins, causing cellular and tissue damage. Furthermore, excessive ROS activates proinflammatory transcription factors for proinflammatory cytokines and adhesion molecules. Compromised endothelial cells attract monocytes that augment inflammation, fostering macrovascular and microvascular injury. ROS has also been associated with epigenetic modifications involved in the maintenance of chronic proinflammatory response [17].

Direct measurement of ROS is challenging due to high biochemical instability. In the last decade, a method for measuring reactive oxygen metabolites (ROMs) in peripheral blood has been developed. This method is the derivatives of the reactive oxygen metabolites (d-ROMs) test, which uses the free radical analytical system. The main component of ROMs is hydroperoxide. Serum levels of hydroperoxides can be measured because of their moderate stability [18].

In the gingiva of subjects with diabetes and periodontitis, markers for increased oxidant stress have been reported; oxidant stress in the gingival tissues can lead to more frequent and more severe periodontal tissue destruction episodes in diabetic patients [19]. Additionally, the chronic release of ROS in the periodontal lesion may diffuse in the bloodstream, threatening the clinical course of other inflammatory systemic diseases [20]. Therefore, increased oxidative stress in patients affected by periodontitis would not be confined to the periodontium; systemic consequences of an increased oxidative burden have been detected in patients with periodontitis, providing a way for periodontal inflammation to worsen glycemic control [21].

The d-ROMs test determines the concentration of hydroperoxides in the blood. The plasmatic antioxidant test (PAT) determines the concentration of water-soluble antioxidants in the blood that are able to reduce ferric ions to ferrous ions. The salivary antioxidant test (SAT) evaluates the salivary total antioxidant capacity [18].

The primary goal of the present randomized clinical study is to investigate the role of non-surgical periodontal treatment in systemic and salivary oxidative stress and glycemic control in diabetic patients with chronic periodontal disease. A secondary aim is to compare different periodontal domiciliary maintenance therapies in terms of periodontal parameters and oxidative stress reduction.

## 2. Materials and Methods

### 2.1. Sample Size Calculation

The sample size was calculated using statistical software and based on the plasma levels of reactive oxygen metabolites (ROMs) from a previous prospective study with a similar design [22]. A sample size of 45 subjects was required for the detection of a significant difference in plasma levels of ROM (80% power, two-sided 5% significance level) before and after non-surgical periodontal treatment. A possible 10% drop-out rate was taken into account.

### 2.2. Subject Recruitment

This study was conducted in full accordance with the World Medical Association Declaration of Helsinki. This study was undertaken with the understanding and written consent of each participant and according to the above-mentioned principles. The patient cohort was derived from a database search at the Tuscan Institute of Stomatology following the inclusion and exclusion criteria outlined below.

### 2.3. Inclusion

The inclusion criteria included:

All patients who had non-surgical periodontal treatment;

All patients who were diabetic (type I or type II);

All patients who had a minimum mid-term follow-up (≥6 months);

All mid-term follow-ups demonstrable by a clinical examination and periodontal recording with periodontal disease [10];

All mid-term follow-ups demonstrable by laboratory measures of HbA1c levels and oxidative stress.

### 2.4. Exclusion

The exclusion criteria included:

All patients who were smokers or alcohol or drugs abusers (more than 20 cigarettes/day);

All patients with psychiatric problems that could contraindicate the oral prognosis;

All patients who received antibiotics within three months before the study started;

All patients who were pregnant;

All patients who had been irradiated to the head or neck in the last 12 months;

All patients who had received intravenous bisphosphonate therapy within 12 months before the study started [23].

### 2.5. Procedure

All the patients received modified full-mouth scaling and full-mouth disinfection using an ultrasonic device and standard periodontal curettes with no time limitation and were prescribed domiciliary adjunctive therapy. The ultrasonic device used was the Mectron Multipiezo device (Mectron s.p.a.). Motivation sessions for proper oral hygiene lasted for 30 to 60 min per patient; the operator informed each patient about the correct maneuvers to perform at home in order to achieve the best results in terms of personal oral hygiene.

All treatment procedures at the hospital were documented in the electronic health records (Quaderno Elettronico s.r.l. (Ar)) or physical dental charts retained at the hospital.

The following analysis comprised three recall visits after baseline: one month (T1), three months (T2), and six months after periodontal treatment (T3). Clinical and laboratory parameters were collected at each of the four time points. Each visit (baseline included) consisted of a full periodontal examination and the collection of saliva and digital blood samples in order to evaluate specific inflammatory markers of oxidative stress as this was the normal practice in the center in which the study took place for diabetic patients. HbA1c levels were registered at baseline, T1, and T3.

### 2.6. Outcome Assessment

The primary study outcome was the change in reactive oxygen metabolite levels from baseline to three months. Secondary outcomes included changes in oxidative stress balance and clinical periodontal parameters from baseline to three months and from baseline to six months.

The null hypothesis was that there would be no differences in terms of reactive oxygen metabolites between baseline and after-treatment values among different groups of treatment.

### 2.7. Periodontal Assessment

One investigator performed full periodontal examinations. The periodontal probing depth (PPD) was measured using a UNC-PCP15 (Hu Friedy) probe at six sites (mesiobuccal, mid-buccal, distobuccal, mesiolingual, and distolingual) per tooth, with the third molars excluded. Mean values of PPD per patient were calculated. Sites that bled upon gentle probing were recorded, and the percentage of total bleeding on probing (BoP) was calculated for each subject [24]. The presence of plaque was assessed dichotomously (“1” present, “0” absent) using the plaque index (PI) at six sites per tooth and then expressed as a percentage of positive sites per patient [25].

### 2.8. Laboratory Analysis

Blood samples were taken chair-side from the fingertip within a few minutes between 10:00 a.m. and 12:00 p.m. and were immediately kept on ice and centrifuged at 3000× *g* for 5 min. The plasma samples (approximately 100 μL per individual) were used to determine plasma levels of reactive oxygen species and antioxidant capacity. Samples were processed according to instructions from the producer (H&D s.r.l.).

The d-ROMs test essentially determines the concentration of hydroperoxides (ROOHs) in the blood, which are substances that belong to a broad class of reactive oxygen metabolites (ROMs) [26]. The d-ROMs test is a photometric test that can be performed on whole blood (generally finger-prick capillary blood, but also venous blood), serum, heparinized plasma, and certain extracellular fluid samples. Its unit of measurement is the U CARR (0.08 mg/dL of a solution of hydrogen peroxide).

The mean standard values for d-ROMs in the normal population, according to the literature using the mentioned measurement system (H&D s.r.l.), range between 250 and 320 U CARR; higher levels are directly proportional to high levels of oxidative stress and inflammation [26].

The plasmatic antioxidant test (PAT) determines the concentration of water-soluble antioxidants in the blood that are able to reduce ferric ions to ferrous ions (U CARR). The PAT is a photometric test that is performed using a photometer in order to determine the antioxidant power of plasma. The mean standard PAT values in the normal population, according to the literature using the mentioned measurement system (H&D s.r.l.), range between 2000 and 2800 U CARR [26]. Saliva samples were taken chair-side after a night of fasting between 07:00 a.m. and 08:00 a.m., with patients kept from washing with mouth rinse. The salivary antioxidant test (SAT) evaluated the salivary total antioxidant capacity, with vitamin C μmol/L as its unit of measure. The SAT test can evaluate salivary AO capacity based on a saliva sample’s ability to reduce ferric ions (Fe^3+^) to ferrous ions (Fe^2+^). The mean standard SAT values in the normal population, according to the literature using the mentioned measurement system (H&D s.r.l.), range between 1500 and 2000 U CARR [26].

Saliva was immediately analyzed since saliva degenerates quickly, altering the absorbance properties of a sample.

### 2.9. Randomization and Allocation

**Figure 1:**
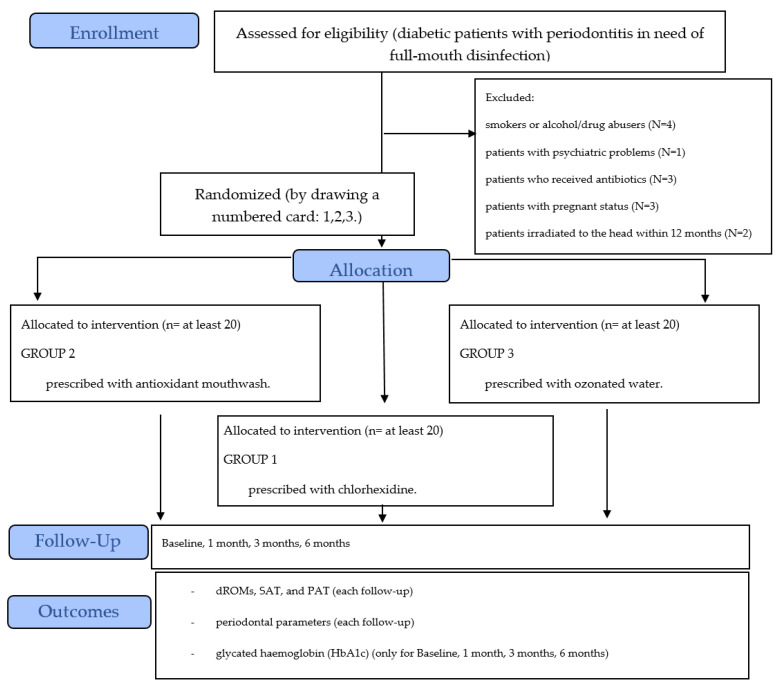
Subject recruitment and treatment allocation flow chart.

After non-surgical periodontal treatment, patients (*n* = 60) were randomly distributed to one of three possible domiciliary treatment groups (20 patients per group) by drawing a numbered card (1, 2, or 3), as described in the flow chart (Figure 1). Clinicians and patients were not blinded because the motivation session was performed according to the allocation. Only the statistician was blinded.

### 2.10. Group 1 or the Chlorhexidine Group (n = 20)

At baseline, patients were given instructions for the domestic use of a chlorhexidine toothpaste (0.20%) and chlorhexidine mouthwash (0.20%) once a day in the evening. The brushing process was performed with either a regular or soft toothbrush in the morning and in the evening [27].

### 2.11. Group 2 or the Antioxidant Group (n = 20)

At baseline, patients were given instructions for the domestic use of a chlorhexidine toothpaste (0.20%) and chlorhexidine mouthwash (0.20%) once a day in the evening for 7 days. The brushing process was performed with either a regular or soft bristle toothbrush in the morning and in the evening. After the first week, patients were given instructions for the domestic use of a lactoferrin-based toothpaste and an antioxidant mouth rinse (toothpaste and mouthwash from Polifarma, Glic^®^) [28].

### 2.12. Group 3 or the Ozone Group (n = 20)

At baseline, patients were given instructions for the domestic use of a chlorhexidine toothpaste (0.20%) and chlorhexidine mouthwash (0.20%) once a day in the evening for 7 days. The brushing process was performed with either a regular or soft bristle toothbrush in the morning and in the evening. After the first week, teeth and gums were brushed using the manual toothbrush with just water, but patients were given instructions for the domestic use of a domiciliary ozone delivery device twice a day before and after brushing their teeth [29].

### 2.13. Data Extraction

A calibrated review identified the cases meeting the study criteria. The self-reported patient demographic data were age, gender, hypertension (yes/no), diabetes mellitus type (I/II), diabetes complications (yes/no), domiciliary home care prescription (chlorhexidine mouth rinse, ozone delivery device, antioxidant mouth rinse). Additionally, periodontal and laboratory measures were retrieved for each patient.

### 2.14. Statistical Analyses

Descriptive and statistical analyses of data were performed with a statistical tool package (R 3.3.3; R Development Core Team, http://www.r-project.org (accessed on 31 March 2018)). The normal distribution was tested with the Shapiro–Wilk method. Baseline values for the variables of interest were tested for homoscedasticity. Outcome variables were defined as the mean probing depth (PPD), percentage of sites with plaque (PI), percentage of sites with bleeding on probing (BOP), mean ROMs, mean PAT, mean SAT, and mean HbA1c. For each variable of interest, changes between baseline and follow-up visits were computed. All measurements in the text and tables are described as means and standard deviations (mean ± SD). Clinical and biochemical parameters were analyzed using the Brunner and Longer model for non-parametric longitudinal analysis. A *p*-value of < 0.05 was considered statistically significant.

## 3. Results

A total of 75 type 1 and type 2 diabetes patients were screened; 11 patients were excluded based on their medical history and 4 for personal reasons, leaving a total of 60 patients eligible for study participation. Table 1 reports the demographic characteristics of the initial cohort. The cohort comprised 22 females and 38 males, with a mean age of 60.9 ± 14.8 years. The mean body mass index was 27.8 ± 14.1, which was positively correlated with the mean amount of plasmatic free radicals at baseline.

Twenty-one out of sixty patients suffered from DM type 1 (seven per group), while the remaining 39 patients were affected by DM type 2. Post-hoc analysis found no significant differences between the two diabetes types for any of the population description factors (sex, number of drugs used, BMI) except for mean age, which was a little lower in patients suffering from diabetes type I. Baseline values for periodontal indices (PPD, PI, BoP) and for plasma and saliva parameters were equally distributed among the three groups of treatment.

### 3.1. Periodontal Findings

**Figure 2:**
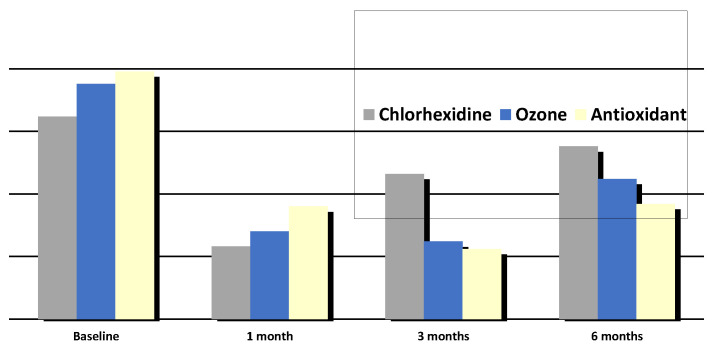
The BoP values decreased for all groups evaluated et each time point, with significant reductions after 1 and 3 months (T1 and T2), values are reported in Table 2.

Table 2 reports the periodontal findings for each group of treatment at every moment of the follow-up. All patients showed statistically significant improvements in all periodontal indices (PPD, PI, BoP) at 1- and 3-month evaluations. All of the treatment groups resulted in comparable efficacy in terms of improvements in clinical parameters. Both the treatment test groups (ozone and antioxidant adjunctive therapy) showed better clinical efficacy than chlorhexidine at the 3-month evaluation, showing statistical significance for a reduction in bleeding sites (BoP), as Figure 2 highlights. However, for all treatment groups, at the 6-month evaluation, significant worsening of the periodontal health status was observed, with all periodontal indices (PPD, PI, BoP) returning to pathological values comparable to those at baseline.

### 3.2. Oxidative Stress Findings

Table 3 reports the ROM and HbA1c outcome measures at each time point of the follow-up. At baseline, the mean plasmatic ROM value for the cohort was 388 ± 21.36 U CARR, which was higher than the mean values for the general population (250–300 U CARR). The ROM dropped significantly at T1, reaching 323 ± 29.19 U CARR after periodontal treatment (*p*-value < 0.05). This finding corroborated the idea of periodontal treatment being able to decrease systemic oxidative stress. At the 6-month evaluation (T3), the mean ROM was 332 ± 31.76 U CARR, denoting a significant relapse towards pathological ranges. No statistical differences were observed among the groups at each time point.

The SAT test showed higher results than normal values at baseline for the entire cohort of patients, indicating the presence of an inflammatory condition. The ozone therapy and the antioxidant mouthwash led to a statistically significant reduction in SAT at the 3-month evaluation compared to the chlorhexidine group. The control treatment did not affect the salivary antioxidant capacity in a significant manner. At the 6-month evaluation, a worsening of SAT test results was observed for all groups.

Similar to the previous biochemical parameters, at baseline, the PAT test also showed abnormal values, indicating the presence of an inflammatory condition. Increases in PAT were observed in all groups at each time point, highlighting reductions in inflammation.

At baseline, the mean HbA1C was 7.18 ± 0.92 for the entire cohort of patients, while regression analysis showed that glycated hemoglobin was related positively to periodontal status at the same time point (*p*-value = 0.05). After three months, HbA1C dropped down to 6.63 ± 0.52 (∆ = −0.52). There were no significant differences among treatment groups in reducing glycated hemoglobin after 3 and 6 months.

A positive correlation was found between the SAT test and PI at baseline for the entire cohort. Furthermore, a positive correlation was found between the mean reduction of glycated hemoglobin and the mean reduction of the pocket probing depth three months after non-surgical periodontal treatment (*p*-value = 0.0176).

## 4. Discussion

In the present clinical study, we assessed the levels of oxidative stress in patients suffering from both diabetes and periodontal disease. To the best of our knowledge, this is the first study evaluating plasma levels of reactive oxygen metabolites in diabetic patients with chronic periodontal disease before and after non-surgical periodontal treatment. The plasma ROM level is considered a reliable indicator of oxidative stress and has been positively correlated with the severity of periodontal disease [30]. At baseline, the cohort of diabetic patients showed a mean ROM level of 388 ± 21.36, which indicated a condition of oxidative stress. Mean baseline PAT values showed a status of slight antioxidant deficiency, the result being coherent with previous studies describing lower levels of blood antioxidants in patients with periodontal disease than in patients with a healthy periodontium [30]. All of the patients included were affected by chronic periodontal disease, with a mean baseline SAT of 2383 ± 360, which would be considered a high value, depicting an overproduction of antioxidant agents towards local infection [31,32]. The results of the present study are in agreement with the literature, relating high levels of salivary antioxidants (SATs) to a possible periodontal inflammatory condition [33,34]. Saliva is easy to collect, in contrast to blood sampling [35]. Stimulation may increase the flow of GCF, which is the main problem when analyzing the concentrations of antioxidants in saliva because an increase in the flow rate may lead to false increases in SAT. However, many diabetic patients (mostly women) show severe xerostomia. The prevalence of drug-induced xerostomia was not calculated because it was not a specific aim of the study.

At the 1-month evaluation (T1), periodontal indices of inflammation (PPD, PI, BoP) decreased significantly, suggesting an improvement in periodontal health after non-surgical therapy. However, PPD and PI did not reach statistically significant differences for the whole-plot factor (group of treatment), indicating that the null hypothesis of no treatment effect on periodontal status could not be rejected. The ozone and antioxidant groups showed the best results in terms of reduction of bleeding on probing, in line with other studies [36].

The oxidative balance of the entire cohort improved significantly after non-surgical periodontal treatment. It is known that oxidative stress, in particular, the oxidation of lipoproteins, is a strong risk factor for cardiovascular diseases [13]. Periodontal treatment offers beneficial effects on systemic health, as periodontitis causes low-grade chronic inflammation and oxidative stress [37].

In 2009, Tamaki and co-workers found a greater mean reduction of plasma ROMs in periodontitis patients (−103 U CARR) after non-surgical periodontal treatment at 1-month evaluation [22]. Regardless, it must be highlighted that their patients did not suffer from diabetes, which is a condition predisposing sufferers to oxidative stress independently from the presence of periodontal disease. In a recent study by Arana and co-workers, salivary oxidative stress levels were measured in patients with diabetes mellitus type 2 (DM2) and healthy non-diabetic patients to investigate whether this oxidative stress was associated with the presence of periodontal disease [38]. Authors found that poor metabolic control in DM2 patients was associated with higher levels of salivary oxidative stress and worse periodontal health.

In the present study, three months after treatment, HbA1 decreased significantly by −0.52 percentage points. This reduction was coherent with the results shown in the systematic review by Engebretson and co-workers in 2013, which reported a mean reduction of −0.36 units 3 months after non-surgical periodontal treatment [39].

Oxidative stress leads to the upregulation of proinflammatory pathways implicated in the pathogenesis of both periodontitis and diabetes [40,41]. A fundamental meta-analysis suggested that periodontitis may be an independent risk factor for coronary heart disease [42]. Recently, in their cross-sectional case–control study, Al-Rawi and Shahid measured salivary levels to indicate lipid profiles, lipid peroxidation, and antioxidant statuses, along with lactate dehydrogenase (LDH), C-reactive protein (CRP), and periodontal statuses in patients with acute coronary heart disease [43]. The authors concluded that periodontal health status could be considered an independent risk factor for acute coronary heart disease (CHD). It would be important to observe longitudinally diabetic patients treated for periodontal disease (SRP and antibiotics prescription) and to check for the incidence of cardiovascular complications. However, the literature suggests that there are no differences between diabetic patients treated with SRP alone or SRP with antibiotics in terms of HbA1c reductions [44]. Therefore, it could be important to identify alternatives to standard non-surgical periodontal treatment protocols for diabetic patients, respecting their altered biochemical pathways. Furthermore, the presence of pathogen bacteria, impaired host resistance, altered collagen synthesis, and oxidative stress makes the diabetic patient more susceptible to periodontal inflammation [45]. In this scenario, as suggested by the results of the present study, the use of an antioxidant mouth rinse or medical ozone delivery device could be integrated with the traditional approach towards periodontal disease, as at the 3-month evaluation, statistically significant differences were appreciated in both test groups when compared with the chlorhexidine control group in terms of BoP reduction. This finding has an important biological rationale; in fact, BoP is better related to present inflammation than PPD, which relates to the progression of periodontal disease in the past.

At the 6-month evaluation, a significant worsening of the periodontal health status was observed in comparison with 1- and 3-month evaluations, with all of the indices explored returning to pathological values comparable to those at baseline. The same trend was observed for systemic and salivary inflammatory markers. Those results might mean that there could be a “periodontal health deadline” for diabetes patients that is definitely shorter than that of healthy patients. The clinical implication of this finding might be important, suggesting the need to redefine treatment guidelines for systemically compromised patients. It is remarkable that in the literature, there is still great debate regarding the relationship between diabetes and periodontal disease, with some studies denying a correlation between diabetes prevalence and the extent or severity of periodontitis [46].

The present authors recommend markers of oxidative stress as potential targets for monitoring the clinical risk of complications in diabetic patients.

## 5. Conclusions

The findings from the present study suggest that non-surgical periodontal treatment might help in reducing systemic inflammation in diabetic patients. Dedicated antioxidant domiciliary oral formulae could be prescribed to diabetic patients as there is mild evidence of their enduring efficacy compared to chlorhexidine. Oxidative stress should be further investigated as an adjunctive marker of risk for the occurrence of complications in diabetic patients presenting periodontal disease.

## Figures and Tables

**Table 1:** Demographic characteristics of the included patients.

Variable	Mean ± SD
No. of patients	60
No. of DM-1	21
No. of DM-2	39
No. of males/females	38/22
No. of smokers	11
Mean age	60.9 ± 14.8 years
Mean body mass index	27.8 ± 0.90

**Table 2:** Mean values (±standard deviations) of clinical periodontal measures at baseline and at one, three, and six months.

Variables	Time Points	Overall		Treatment Groups	
			*Chlorhexidine*	*Ozone*	*Antioxidant*
		Mean ± SD	Mean ± SD	Mean ± SD	Mean ± SD
**PPD**	*Baseline*	3.13 ± 0.36	3.13 ± 0.35	3.16 ± 0.33	3.09 ± 0.39
	*1 month*	2.09 ± 0.19	2.09 ± 0.12	2.07 ± 0.22	2.11 ± 0.21
	*3 months*	2.03 ± 0.21	2.35 ± 0.39	2.02 ± 0.18	1.98 ± 0.22
	*6 months*	2.10 ± 0.21	2.11 ± 0.19	2.09 ± 0.21	2.08 ± 0.19
**%PI**	*Baseline*	0.85 ± 0.19	0.83 ± 0.17	0.81 ± 0.21	0.89 ± 0.26
	*1 month*	0.15 ± 0.05	0.13 ± 0.09	0.15 ± 0.06	0.17 ± 0.05
	*3 months*	0.35 ± 0.14	0.33 ± 0.12	0.34 ± 0.18	0.37 ± 0.09
	*6 months*	0.63 ± 0.07	0.65 ± 0.15	0.53 ± 0.13	0.64 ± 0.18
**%BoP**	*Baseline*	0.96 ± 0.11	0.81 ± 0.18	0.94 ± 0.11	0.99 ± 0.10
	*1 month*	0.41 ± 0.12	0.29 ± 0.18	0.35 ± 0.10	0.45 ± 0.14
	*3 months*	0.31 ± 0.16	0.58 ± 0.15	* 0.31 ± 0.17	* 0.28 ± 0.24
	*6 months*	0.64 ± 0.13	0.69 ± 0.19	0.56 ± 0.17	0.46 ± 0.18

* The mean values in bold are significantly different from the chlorhexidine group at *p* < 0.05.

**Table 3:** The outcome measures for reactive oxygen metabolites (dROMs, PAT, SAT) and glycated hemoglobin (HbA1c) were measured at baseline and at one, three, and six months.

Variable	Time Point	Overall		Treatment Group	
			*Chlorhexidine*	*Ozone*	*Antioxidant*
		Mean ± SD	Mean ± SD	Mean ± SD	Mean ± SD
**dROMs**	*Baseline*	388 ± 21.36	373 ± 27.15	399 ± 30.23	385 ± 24.34
	*1 month*	323 ± 29.19	320 ± 23.24	328 ± 31.12	327 ± 27.26
	*3 months*	294 ± 34.21	329 ± 18.29	288 ± 40.18	291 ± 28.56
	*6 months*	332 ± 31.76	336 ± 41.29	331 ± 24.19	332 ± 34.85
**PAT**	*Baseline*	1883 ± 165	1880 ± 98.5	1847 ± 21.7	1819 ± 54.4
	*1 month*	1961 ± 131	1991 ± 85.9	1964 ± 79.7	1922 ± 45.4
	*3 months*	2002 ± 152	1977 ± 185	2346 ± 73.9	2264 ± 81.3
	*6 months*	2076 ± 143	1907 ± 27.5	2258 ± 89.6	2060 ± 77.7
**SAT**	*Baseline*	2383 ± 360	2157 ± 241	2347 ± 636	2459 ± 874
	*1 month*	1751 ± 215	1573 ± 343	1827 ± 696	1969 ± 179
	*3 months*	1687 ± 396	1927 ± 402	* 1625 ± 278	* 1519 ± 477
	*6 months*	1971 ± 715	2173 ± 697	1827 ± 596	1969 ± 179
**HbA1c**	*Baseline*	7.18 ± 0.92	7.10 ± 0.81	7.27 ± 0.72	7.22 ± 1.03
	*3 months*	6.63 ± 0.52	6.71 ± 0.55	6.58 ± 0.69	6.60 ± 0.80
	*6 months*	6.94 ± 0.33	7.01 ± 0.29	6.86 ± 0.19	6.96 ± 0.23

* The mean values in bold are significantly different from the chlorhexidine group at *p* < 0.05.

## Data Availability

Additional data may be available if requested from the authors at Tuscan Stomatologic Institute. The data are not publicly available because the clinical study is still going on with longer follow-up.
